# Outbreak of *E. coli* O157:H7 Infections Associated with Exposure to Animal Manure in a Rural Community — Arizona and Utah, June–July 2017

**DOI:** 10.15585/mmwr.mm6723a2

**Published:** 2018-06-15

**Authors:** Sarah Luna, Vikram Krishnasamy, Louise Saw, Lori Smith, Jennifer Wagner, Jenna Weigand, Mackenzie Tewell, Marilee Kellis, Roumen Penev, Laine McCullough, Jeffrey Eason, Keegan McCaffrey, Cindy Burnett, Kelly Oakeson, Melissa Dimond, Allyn Nakashima, Deidre Barlow, Anna Scherzer, Melanie Sarino, Morgan Schroeder, Rashida Hassan, Colin Basler, Matthew Wise, Laura Gieraltowski

**Affiliations:** ^1^Epidemic Intelligence Service, CDC; ^2^Utah Department of Health; ^3^Utah Public Health Laboratory; ^4^Southwest Utah Public Health Department; ^5^Arizona Department of Health Services; ^6^Mohave County Department of Public Health, Arizona; ^7^La Paz County Health Department, Arizona; ^8^Caitta, Inc., Herndon, Virginia; ^9^Division of Foodborne, Waterborne, and Environmental Diseases, National Center for Emerging and Zoonotic Infectious Diseases, CDC.

On June 26, 2017, a hospital in southern Utah notified the Utah Department of Health of Shiga toxin–producing *Escherichia coli* (STEC) O157:H7 infections in two children from a small community on the Arizona-Utah border. Both children developed hemolytic uremic syndrome, characterized by hemolytic anemia, acute kidney failure, and thrombocytopenia and died within a few days of illness onset. Over the next few days, several more STEC-associated illnesses were reported in residents of the community. A joint investigation by local and state health agencies from Arizona and Utah and CDC was initiated to identify the outbreak source and prevent additional cases; a total of 12 cases were identified, including the two children who died. Investigators initially explored multiple potential sources of illness; epidemiologic and environmental information revealed cow manure contact as the likely initial cause of the outbreak, which was followed by subsequent person-to-person transmission. One of the outbreak strains was isolated from bull and horse manure collected from a yard near a community household with two ill children. Local health agencies made recommendations to the public related to both animal contact and hand hygiene to reduce the risk for STEC transmission. Animal or animal manure contact should be considered a potential source of STEC O157:H7 during outbreaks in communities where ruminants are kept near the home.

## Epidemiologic Investigation

A case of STEC O157:H7 infection was defined as an illness in a resident of the Centennial Park/Colorado City/Hildale community with onset of diarrhea after June 1, 2017, with 1) culture-confirmed STEC O157:H7 with one of three novel pulsed-field gel electrophoresis (PFGE) pattern combinations or 2) physician-diagnosed postdiarrheal hemolytic uremic syndrome. Cases were classified as secondary if contact with another case occurred ≥3 days before illness onset. Local health care facilities identified potential cases via syndromic surveillance and reported them to the Southwest Utah Public Health Department and the Mohave County (Arizona) Health Department. The Southwest Utah Public Health Department created several social media posts advising community residents with diarrhea to see a doctor because local health officials were concerned that adults in this community would not seek health care for themselves.

**FIGURE 1:**
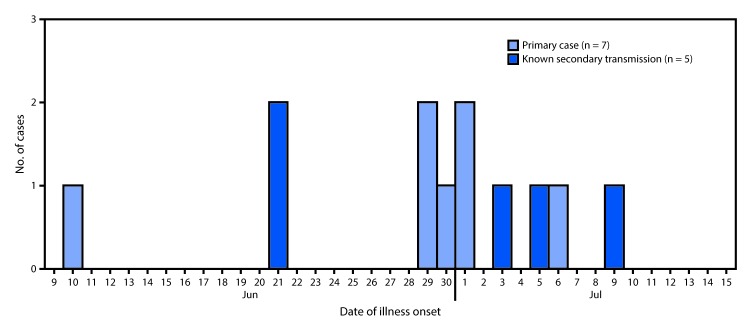
Number of cases of Shiga toxin–producing *Escherichia coli* O157:H7 infection, by date of illness onset — Centennial Park/Colorado City/Hildale community, Arizona and Utah, June–July 2017

Twelve cases were identified, including five classified as secondary, from eight separate households. Illness onset dates for the 12 patients ranged from June 10 to July 9, 2017 (Figure 1). The median age of patients was 3 years (range = 1–28 years), and 11 were aged ≤6 years. Five cases occurred in females; nine patients were hospitalized, four had hemolytic uremic syndrome, and two died.

All patients or their guardians were interviewed using a hypothesis-generating questionnaire containing questions about foods eaten, food source locations, travel, recreational water exposure, sources of drinking water, and animal contact during the week before illness onset. All 12 patients or their guardians reported shopping at grocery store A, and guardians of six of seven patients with primary cases reported purchasing ground beef. The prevalence of ground beef consumption was significantly higher than that reported in the Foodborne Diseases Active Surveillance Network Population Survey (FoodNet; https://www.cdc.gov/foodnet/index.html) (86% versus 40%; p = 0.04) (*1*); however, local health officials suspected a higher typical ground beef consumption rate in this community than in the nation overall. Thus, other potential hypotheses were explored in a focus group discussion with five guardians of four ill children. Beef and watermelon consumption, contact with domestic and companion animals, and multiple exposures to recreational water emerged as common exposures.

A 1:3 matched case-control study was designed based on information from the focus group discussion. Guardians of 16 healthy children were recruited through an online survey posted to a closed Facebook group of current and past community residents. The voluntary survey included screening questions to determine their children’s eligibility for participation. Community health workers used a focused questionnaire containing questions about consumption of ground beef and fresh produce, as well as all animal contact during the exposure period to interview the guardians of six of seven patients with primary cases and guardians of 16 healthy age-matched controls. Four of six ill children and three of 16 controls reported playing in an area that had animal manure (matched odds ratio = 7.7; 95% confidence interval = 0.8–71.3) (Table).

**TABLE:** Number of exposures to selected food, water, and animals, and matched odds ratios comparing patients with primary cases of Shiga toxin–producing *Escherichia coli* O157:H7 infection (n = 6) with healthy children (n = 16) — Centennial Park/Colorado City/Hildale community, Arizona and Utah, June–July 2017

Exposure	Case-patients no. (%)	Controls no. (%)	Matched odds ratio (95% CI)
Played in area with animal manure	4 (67)	3 (19)	7.7 (0.8–71.3)
Touched cow	2 (33)	1 (6)	5.3 (0.5–58.7)
Dogs wandered on property	4 (80)	7 (44)	4.1 (0.4–38.0)
Drank municipal water	3 (50)	3 (19)	3.1 (0.5–19.3)
Swimming	5 (83)	10 (63)	2.4 (0.3–21.3)
Consumed beef prepared at home	3 (50)	12 (75)	0.3 (0.03–2.8)
Consumed watermelon*	5 (100)	10 (63)	—

**Abbreviation:** CI = confidence interval.

* Only five of the six case-patients responded to the question on watermelon.

**FIGURE 2:**
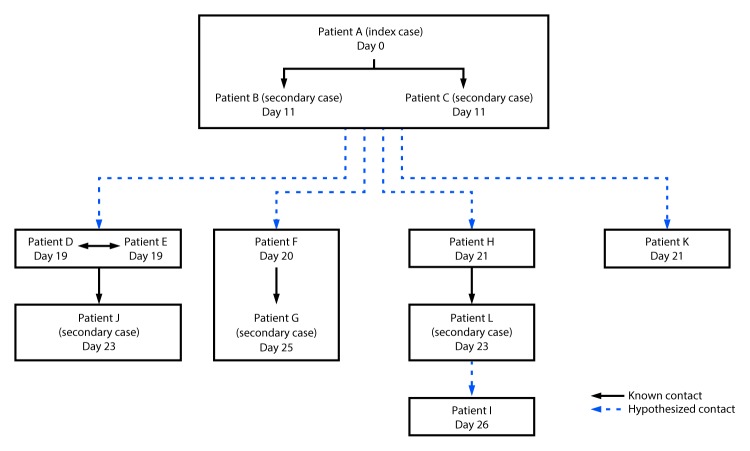
Number of cases of Shiga toxin–producing *Escherichia coli* O157:57 infection, by type of case and numbered day in the outbreak — Centennial Park/Colorado City/Hildale community, Arizona and Utah, June–July 2017* * Boxes represent households.

Contact tracing identified friendships, working relationships, or familial relationships between persons in all eight households. Illness onset dates were consistent with hypothesized person-to-person contact (Figure 2). The three patients with the earliest illness onset dates (patients A, B, and C), including the two patients who died, lived in the same multifamily household with approximately 40 persons. After the second patient died, the house was voluntarily vacated, and many persons moved within the community. Contact with animal manure was the hypothesized source of the initial illnesses, with further spread via secondary person-to-person transmission.

## Laboratory Investigation

Officials from the Utah Department of Health and the Mohave County (Arizona) Health Department collected food, water, animal feed and manure, and environmental samples from various locations in the community. The Utah Public Health Laboratory and Arizona State Public Health Laboratory tested 143 samples for STEC.

A total of 35 samples from grocery store A included ground beef and environmental samples from the meat grinder, meat preparation areas, and meat storage areas. Officials also collected samples of frozen ground beef from households and samples of animal manure from cattle (23), goats (five), horses (17), dogs (11), and other animals (six) in the Centennial Park/Colorado City/Hildale community. Drinking and recreational water samples (12) were collected from surrounding farms and creeks. Stool specimens were obtained from 11 patients.

STEC was not isolated from any of the food or environmental samples from grocery store A. However, STEC was isolated from the 11 patient specimens and three animal manure samples (two horses and one bull). All isolates were further characterized by whole genome sequencing. Bioinformatic analysis (*2*) performed at the Utah Public Health Laboratory indicated the 11 clinical isolates, one bull manure isolate, and two horse manure isolates formed a single monophyletic clade with short branch lengths and high statistical support based on bootstrap statistical analysis of 1,000 replicates. This finding indicated that all the isolates were highly related genetically and shared a common molecular evolutionary history. High-quality single-nucleotide polymorphism (hqSNP) analysis performed at CDC (*3*) indicated that the 11 clinical isolates, one bull manure isolate, and two horse manure isolates differed by 0–4 hqSNPs, suggesting that they were highly related genetically. STEC O157:H7 was not isolated from samples from the source farms or animal feed.

## Public Health Response

This multijurisdictional investigation involved daily collaboration among national, state, and local agencies facilitated by an incident command structure. Public communication and educational materials were developed by the Southwest Utah Public Health Department and disseminated by investigation partners, including a public health nurse who was a member of the community. Educational information focused on hygiene related to livestock, safe cooking, increased vigilance for gastrointestinal symptoms, and prevention of secondary transmission. No additional STEC cases with the outbreak strain have been reported from this community since the conclusion of the investigation.

## Discussion

In this outbreak, playing in an area with animal manure was associated with illness. The five ill children with the earliest illness onset dates lived in close proximity to one another and the culture-positive animal manure. STEC can be shed intermittently by colonized animals, so additional animals might have carried the outbreak strain despite the lack of isolation from manure. Unlike ruminants, horses are not considered reservoirs for STEC O157:H7 (*4*,*5*). The hypothesis is that the two horses were infected with the outbreak strain while living in proximity to the bull.

This investigation highlights the use of multiple epidemiologic methods, including hypothesis-generating questionnaires, focus group interviewing, a case-control study, and contact tracing in concert with environmental and clinical testing in identifying the source of an outbreak. These methods were used to generate and test hypotheses regarding four modes of disease transmission: person-to-person, food, drinking and recreational water, and animal contact.

This investigation also highlights the importance of communication and outreach efforts to successful, sensitive public health investigations. The inclusion of a local public health nurse in the investigation team enhanced communication and facilitated both the focus group and contact tracing efforts within a community that had been wary of government officials during previous public health interventions.

The findings in this report are subject to at least three limitations. First, this outbreak spread through secondary person-to-person transmission, limiting the number of primary cases available for assessment of exposure frequencies for hypothesis generation. Second, for all methods used to investigate hypotheses, ill children or their guardians were contacted 1–6 weeks after the illness began, which could have resulted in inaccurate recall of food and animal contact. Finally, low health care utilization among members of the adult population might have resulted in unidentified cases. These limitations might have decreased the likelihood of statistically significant epidemiologic findings despite positive identification of the outbreak strain in animal manure.

Based on the epidemiologic and environmental data, it is likely that the initial source of this outbreak was contact with animals or their environments. Certain behaviors in the patients with primary cases might have contributed to initiation of the outbreak, such as lack of awareness of the risk for disease, inadequate hand washing, and hand-to-mouth behaviors. Subsequent person-to-person transmission resulted in a large, severe outbreak that included challenges in identifying the source. Strong multijurisdictional partnerships and a combination of epidemiologic methods were necessary to identify an outbreak source. Promoting adequate sanitation and hand washing practices around animal and manure exposure is critical to prevent future outbreaks.

**Summary**

**What is already known about this topic?**

Ruminants can be reservoirs for Shiga toxin–producing *Escherichia coli* (STEC) O157:H7 infections; these infections often cause severe human illness.

**What is added by this report?**

Twelve cases of STEC O157:H7 infection associated with exposure to animal manure and secondary person-to-person transmission occurred in an Arizona-Utah border community. Bull and horse manure containing the outbreak strain were identified in a yard near that of the first seven patients; contact tracing revealed plausible person-to-person transmission among all patient households.

**What are the implications for public health practice?**

Hand hygiene is important to reduce the risk for STEC O157:H7 transmission. Contact with animals or animal manure should be considered in outbreak investigations when ruminants are kept near the home.

## References

[1] CDC. Foodborne Diseases Active Surveillance Network (FoodNet) population survey atlas of exposure, 2006–2007. Atlanta, GA: US Department of Health and Human Services, CDC; 2008. https://www.cdc.gov/foodnet/surveys/foodnetexposureatlas0607_508.pdf

[2] Oakeson KF, Wagner JM, Mendenhall M, Rohrwasser A, Atkinson-Dunn R. Bioinformatic analyses of whole-genome sequence data in a public health laboratory. Emerg Infect Dis 2017;23:1441–5. 10.3201/eid2309.170416 28820135 PMC5572866

[3] Katz LS, Griswold T, Williams-Newkirk AJ, A comparative analysis of the Lyve-SET phylogenetics pipeline for genomic epidemiology of foodborne pathogens. Front Microbiol 2017;8:375. 10.3389/fmicb.2017.00375 28348549 PMC5346554

[4] Lengacher B, Kline TR, Harpster L, Williams ML, Lejeune JT. Low prevalence of *Escherichia coli* O157:H7 in horses in Ohio, USA. J Food Prot 2010;73:2089–92. 10.4315/0362-028X-73.11.2089 21219723

[5] Williams AP, McGregor KA, Killham K, Jones DL. Persistence and metabolic activity of *Escherichia coli* O157:H7 in farm animal faeces. FEMS Microbiol Lett 2008;287:168–73. 10.1111/j.1574-6968.2008.01310.x 18715227

